# Supplementary material for: Demonstration of electron focusing using electronic lenses in low-dimensional system
Source: Sci Rep. 2020 Feb 13;10:2593. doi: 10.1038/s41598-020-59453-x (PMC7018971; doi:10.1038/s41598-020-59453-x)
Supplement: Supplementary file 1 — Supplementary Information. [file 41598_2020_59453_MOESM1_ESM.pdf]

Supplementary information: Demonstration of electron focusing using electronic lenses in low-dimensional system

Chengyu Yan,[1,2,3,*] Michael Pepper,[1,2] Patrick See,[4] Ian Farrer,[5] David Ritchie,[6] and Jonathan Griffiths[6]

[1]*London Centre for Nanotechnology, 17-19 Gordon Street, London, WC1H 0AH, United Kingdom*
[2]*Department of Electronic and Electrical Engineering, University College London, Torrington Place, London, WC1E 7JE, United Kingdom*
[3]*Micronova, Aalto University, Tietotie 3, Otaniemi, Espoo, 02150,Finland*
[4]*National Physical Laboratory, Hampton Road, Teddington, Middlesex TW11 0LW, United Kingdom*
[5]*Department of Electronic and Electrical Engineering, University of Sheffield, Sheffield, S1 3JD, United Kingdom*
[6]*Cavendish Laboratory, J.J. Thomson Avenue, Cambridge, CB3 OHE, United Kingdom*

(Dated: January 12, 2020)

I. EXPERIMENT SETUP

The wafer structure is summarized in Table I. The fabrication procedure is briefly listed here: To define the shape of the Hall bar, the chip is coated with a negative photoresist (Shipley 1813) and then exposed to UV light through a mask patterned as a Hall bar. After this, the chip is developed into MF319 for roughly 30 s to remove the photoresist from the exposed area. Therefore, only the hall bar itself is covered by photoresist after this step. Then, the chip is etched with an etching solution (1:8:120 H_2SO_4:H_2O_2:H_2O), the uncovered part is etched out to leave a raised mesa. The Ohmic contacts, made of an alloy of AuGeNi, is deposited via thermal evaporation and annealed in forming gas (95% N_2 and 5% H_2). The split gate defined with standard e-beam lithography. First, a PMMA layer is deposited on the chip and baked at 150° for 60 min. Then the PMMA layer is exposed by the electron beam. The chip is then developed in IPA: MIBK (1:3). After this, a 20/60 nm Ti/Au film is metalized to the chip. Finally, it is lift-off in acetone. The fabrication procedure for the lens gate is rather similar to split gate but with an additional PMMA layer (with a thickness of 400 nm) underneath. After the fabrication, the electron density (mobility) was 1.80×10^{11}cm^{-2} (2.1×10^{6}cm^{2}V^{-1}s^{-1}). The mean free path is around 15 μm.

The measurement is performed with standard lock-in technique, as shown in Fig.S1(a). An ac excitation voltage of 5 μV is applied if both QPC1 and QPC2 are used as injectors (the two QPCs are usually set at the same conductance); or the excitation will be increased to 10 μV if only QPC1 or QPC2 is used. The change in excitation voltage ensures the current injected into the 2DEG is almost the same with both setup.

A red LED is integrated into our sample holder, which is about 1 cm from the cap layer (on which the gates patterned) of the sample. Due to the relatively large distance between the LED and sample, the light spot is with a radius of 100 μm and can cover the entire gated area (both the split gate and lens gate), and hence control the global electron density.

Layer	Thickness	Material
substrate	500 μm	bulk GaAs
buffer	500 nm	GaAs
supperlattice	200 × 5 nm	alternating GaAs/AlGaAs
host	1000 nm	GaAs
spacer	40 nm	AlGaAs
doping	40 nm	Si-doped AlGaAs
cap	10 nm	GaAs

TABLE I. The wafer is grown from substrate to cap layer.

II. COMMENT ON LENS DESIGN

It might be suggested that one can design a lens gate with a double-convex shape to realize electron focusing, hereafter we refer this approach of design as direct-approach; the design presented in the main text is then a complementary version of the direct-approach, and thus named as complementary-approach. With direct-approach, the necessary condition for focusing lens is $N_r = \sqrt{\frac{n_1}{n_2}} > 1$ where n_1 and n_2 are electron density underneath the electrostatically defined lens and that outside lens, respectively, in other words $n_1 > n_2$. A positive lens gate voltage has to be applied to make $n_1 > n_2$. For our specific device, the focusing peak takes place at $N_r \approx$ 1.5, i.e., $n_1 > 2n_2$, which means a large positive lens gate voltage is required. The large positive gate voltage may cause serious leakage between the gate and wafer. Therefore we choose the complementary-approach, where $N_r > 1$ is achieved by reducing n_2 via applying negative gate voltage.

SEM images of the devices can be found in Fig. S1. The dimension of the hollow area is intentionally left large to avoid the formation of a quantum dot. It might be noted that there are some roughness on the surface of the lens gate. The irregular larger spots on the lens gate due to shallow pits in the underlying crosslink PMMA. From our previous experiment, these pits (on PMMA) can have depth up to 20 nm, the total thickness of the PMMA is 400 nm, and hence, we believe isolation is not affected by these pits.

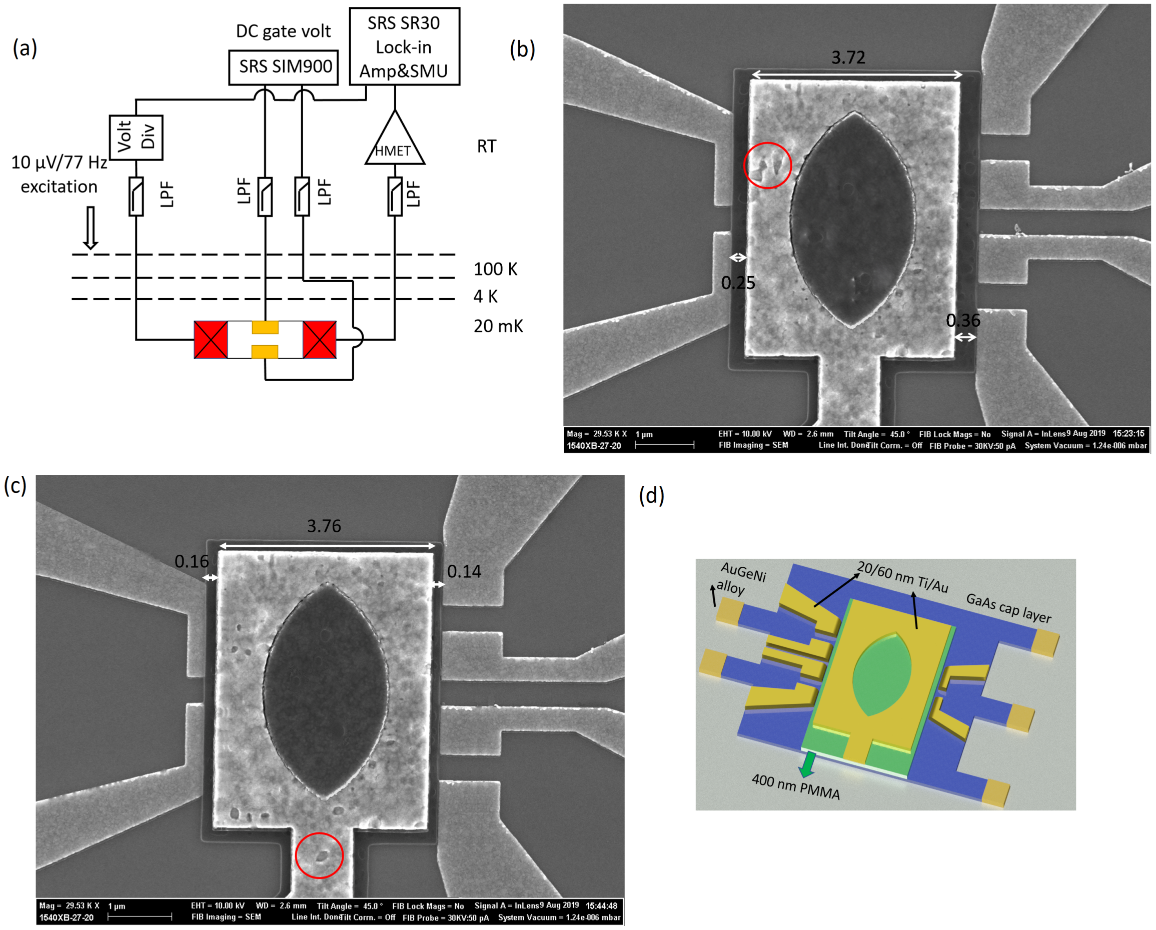

Supplemental Fig 1. (a) Experiment setup. The device layout and inner structure of the refrigerator are simplified. SRS SR30 lock-in amplifier has a built-in source-measurement unit (SMU). (b) and (c) show SEM images of device with 300 nm (device C) and 150 nm gap (device B) between the split gates and lens gate, respectively. Device A, from which we extract most of the data, is the same as the imaged device apart from the 100 nm gap. The lens is offset by ∼50 nm along the primary axis for device C; however, the offset in this direction does not affect the formation of focusing peak. The surface roughness on the lens gate, exampled by the red circle, arises from the irregular shallow pit on the PMMA layer. (d) Schematic representation of the device layout. The squares at the edge of the mesa represent Ohmic contacts. Only the cap layer of the wafer is shown in the layout. The figure is not to scale.

III. DATA FROM DIFFERENT THERMAL CYCLES AND DEVICES

The position of the focusing peak and hence the calculated N_r is robust after different cooling down, as shown in attached Fig. S2(a). The detailed spectrum varies from different cooling down; for instance, the amplitude of the focusing peak decays a bit in second cooling. The peaks in the regime where current drops rapidly seem to be robust in different cooling down.

Although we do not have a device with different lens size, we have performed a brief measurement on another device (dev B) with a larger gap between the lens gate and QPCs, the comparison between dev A (where we extract the main result) and dev B is shown in Fig. S2(b). For device B, the focusing peak occurs at -0.92, and the pinched-off voltage increases to -1.62, inserting these values to lensmaker's equation, it gives a focal length l_f = 2.39 um (the lithographical value is 2.30 um). Also, we can notice that the peaks in the regime where current drops rapidly are noticeably different from those in dev A.

IV. DISCUSSION ON OTHER POSSIBLE ORIGIN OF THE FOCUSING PEAK

It might be concerned that the focusing peak may arise from trivial electrostatic or coherent effects.

The electrostatic effects to be concerned here are the asymmetry in the lens gate design, i.e. only one side of the lens is connected by a metallic arm (Fig. S1), cross-coupling between the split gates and lens gate and charging effect, in other words the focusing peak is related to Coulomb blockade.

I. The asymmetry in the lens gate design. The in-

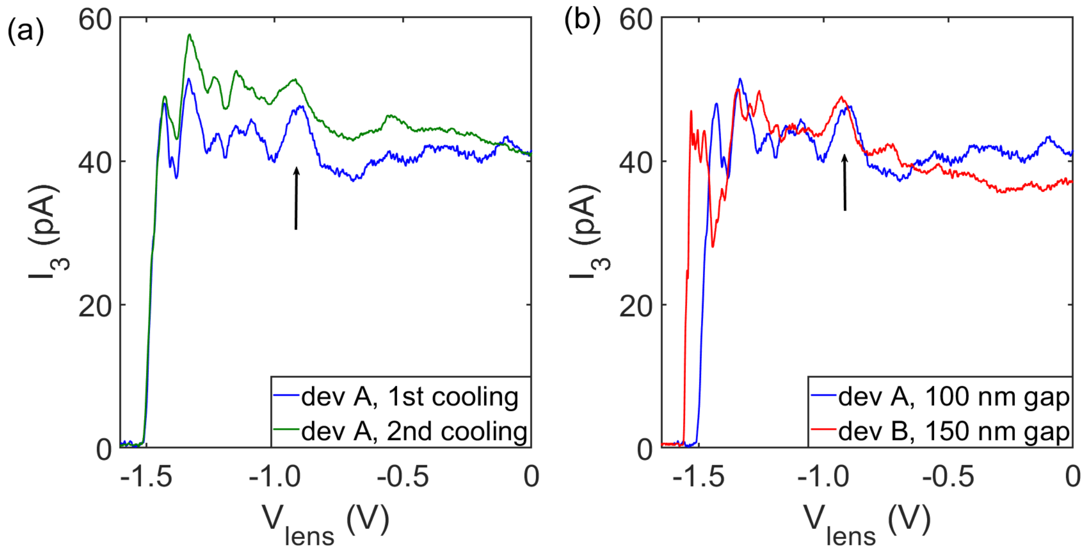

Supplemental Fig 2. Focusing spectrum extracted from (a) different cooling down and (b) different devices.

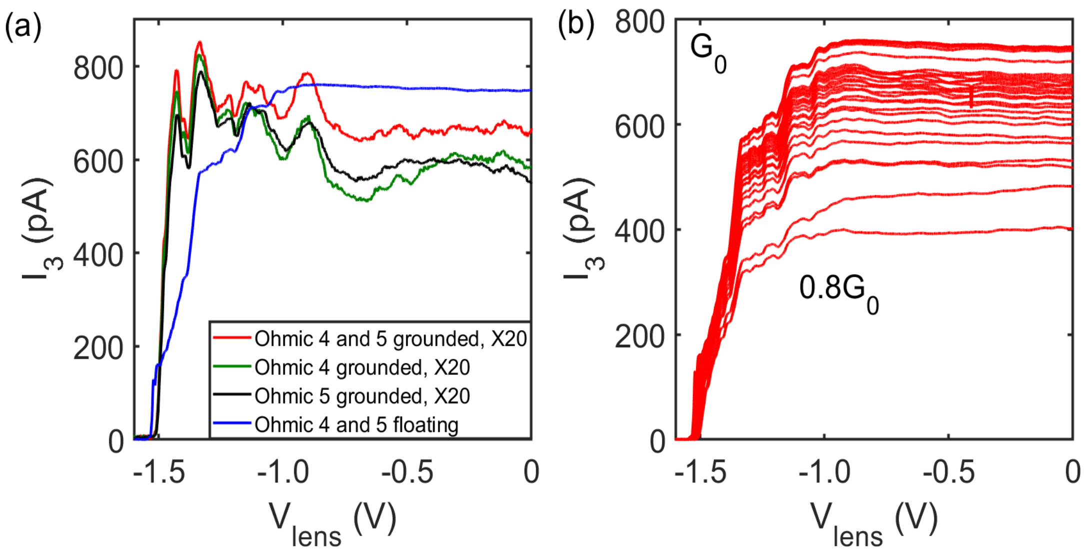

Supplemental Fig 3. Results with different grounding schemes. (a) Comparison between results with both Ohmic 4 and 5 grounded, only Ohmic 4 grounded (green), only Ohmic 5 grounded (black) and both Ohmics floating (blue). Injector and detector were set to G_0. Ohmic 1-3 were always used. (b) Electronic focusing results as a function of injector conductance with both Ohmic 4 and 5 floating. Injector was incremented and detector was set to G_0.

fluence of the asymmetry can be checked by different grounding schemes, i.e. grounding either Ohmic 4 or 5, grounding both or let both of them floating (Ohmic 1-3 are always used). If the asymmetry indeed affects the result, then one would expect that data obtained with only Ohmic 4 grounded should differ from that with Ohmic 5 grounded. The experimental results reveal that the focusing peak always occur if Ohmic 4 or/and 5 is grounded, as depicted in Fig. S3. Hence, it is clear that the asymmetry in the lens gate design does not influence the main result.

II. The cross-coupling between the split gates and lens gate. Two observations suggest the cross-coupling does not affect the focusing peak. First, there is no explicit focusing peak when both Ohmic 4 and 5 are floating as shown in Fig. S3. If the cross-coupling causes the focusing peak, then it should not be affected by the grounding scheme. Instead, the reason why focusing peak is absent when both Ohmic 4 and 5 are floating relies on the fact all the injected electrons have to leave the system through the detector no matter focusing happened or not in the case. Second, the position of focusing peak is ro-

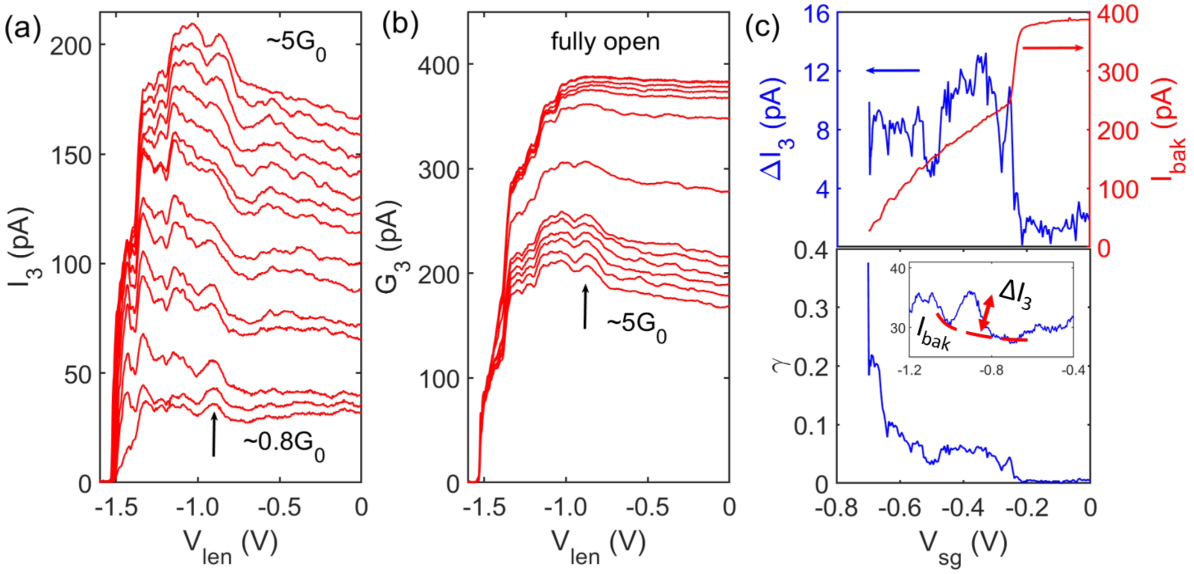

Supplemental Fig 4. Electronic focusing results as a function of injector conductance. QPC 1 and 2, the injectors, were incremented simultaneously from $0.8G_0$ to channel fully open while the detector was fixed at G_0. Ohmic 4 and 5 are grounded. **(a)** Results in regime I ($0.8G_0$ to $5G_0$) where the focusing peak (marked by the black arrow) was initially strong, then significantly weakened when the injector conductance approached $3G_0$ and eventually reappeared. **(b)** Results in regime II ($5G_0$ to channel fully open), the focusing peak smeared out when the injector QPCs entered the 2D regime. **(c)** Intensity of the focusing peak ΔI_3, background signal I_{bak} at V_{lens} = -0.9V (where the maximum of the focusing peak occurred), and focusing efficiency $\gamma = \Delta I_3/I_{bak}$ with respect to the split gate voltage of injector QPC1. ΔI_3 is defined by subtracting a polynomial background (i.e., I_{bak}) from the raw data in the vicinity of the focusing peak as shown by the inset of **(c)**.

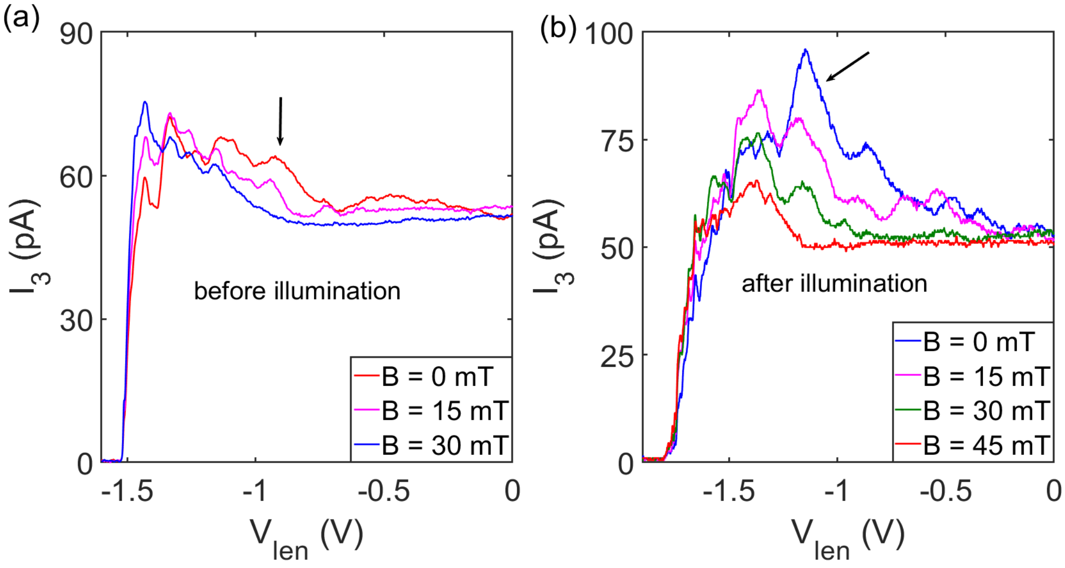

Supplemental Fig 5. Evolution of focusing peak against transverse magnetic field. Injectors and detector was set to G_0. Plot (a) and (b) show the data before and after illumination, respectively. The bold black arrow highlights focusing peak.

bust against the injector conductance as shown in Fig. S4. The split gate voltage would cause an drift in the effective lens gate voltage, and vice versa, in the presence of cross-coupling. Hence, it should expect a shift in the peak position when the split gate voltage (and therefore the conductance) is incremented, if the focusing peak is related to the cross-coupling. This scenario contradicts to the experimental result. Therefore, we conclude that the cross-coupling, if there is any, does not result in the focusing peak.

III. The charging effect, or Coulomb blockade peak. If the focusing peak is related to Coulomb blockade peak as-

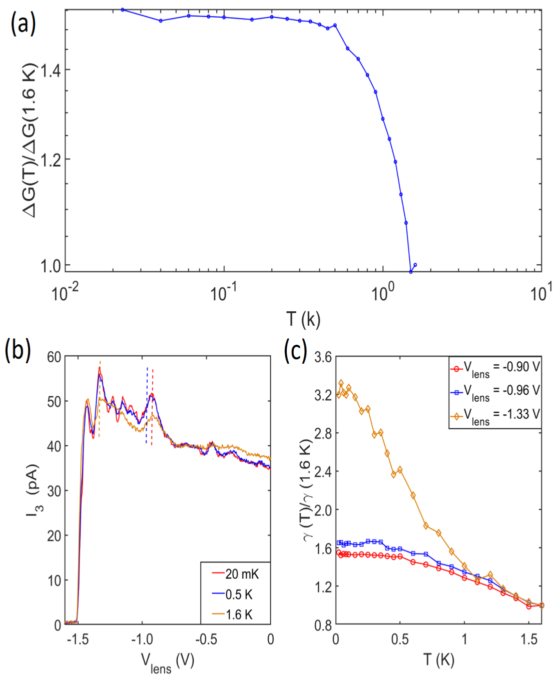

Supplemental Fig 6. Temperature dependence of the focusing peak amplitude. (a) This data is the same as that shown in Fig. 2 of the main text, but with both axis in the log-scale. A linear behaviour, if plotted in this way, is expected if the focusing peak is associated with universal conductance fluctuation. (b) Typical traces at three different temperature. (c) Normalized peak amplitude, after subtracting the background defined by scaling the data with both Ohmic 4 and 5 floating, as a function of temperature at three different lens gate voltage.

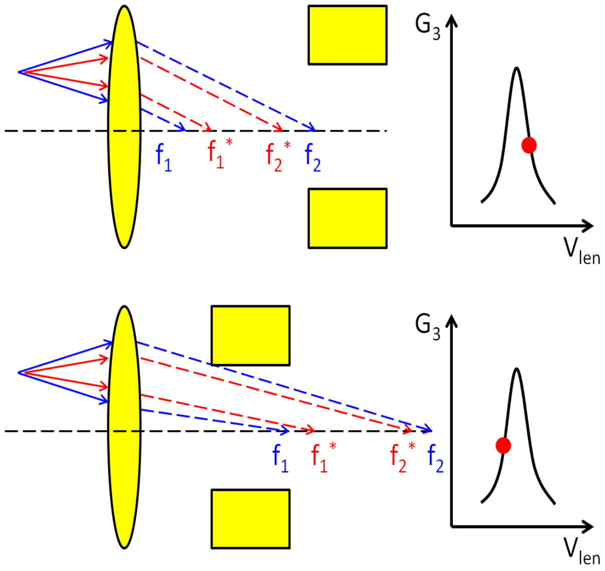

Supplemental Fig 7. Relation between FWHM of focusing peak and angular spread. **(a)** and **(b)** illustrate the spatial distribution of injected electrons on the primary axis with lens gate voltage set to the right and left FWHM point of focusing peak, respectively. f_1 and f_2 are for large angular spread; on the other hand, f_1^* and f_2^* are for smaller angular spread. The double-convex stands for the lens and square blocks form detector QPC.

suming an undesired quantum dot (QD) forms in between the split gates and lens gate, then it should be robust against (small) transverse magnetic field. In our experiments, the focusing peak is highly sensitive to the transverse magnetic field of several 10s mT, see Fig. S5. In addition, the Coulomb blockade peak should be sensitive to the split gate voltage since it influences the size of the undesired QD, however, the focusing peak is rather robust against the split gate voltage (for instance the asymmetric gate biasing shown in Fig. 3 of the main text). On the other hand, the residual peaks in the regime where I_3 drops rapidly are insensitive to the magnetic field but changes dramatically against split gates voltage, thereby we suggests these residual peaks are probably due to the Coulomb blockade.

The coherent effects to be considered here are the universal conductance fluctuation (UCF) and weak localization (WL)/weak anti-localization (WAL).

IV. UCF. UCF in GaAs heterojunction has been widely studied, and it is suggested that the fluctuation ΔG, which in our experiment is the amplitude of the focusing peak, should follow a decay against temperature $\Delta G \sim T^{-a}$. We replot the data in Fig. 2 of the main text, as shown in Fig. S6, to check the validity of this relation. It is seen that the result does not follow the trend especially when the temperature is lower than 800 mK. It might be argued that the discrepancy is because of the saturation of phase coherence length. However, it should be mentioned that the saturation usually occurs around 100 mK or even lower in high quality GaAs sample[1,2], instead of the relatively high temperature of 800 mK observed here. Hence, we think UCF does not count for the focusing peak.

V. WL or WAL. WL or WAL interpretation of the focusing peak can be excluded by the fact that it is not present when Ohmic 4 and 5 are floating. Besides, usually the WAL peak in GaAs heterojunction is with an FWHM less than 1 mT whereas the FWHM of the 'WAL peak' in our device is in the order of 10 mT, which roughly yields a spin-orbit interaction coefficient in the order of 10^{-12} eVm, i.e. at least two orders larger than that expected from GaAs heterojunction even with considerable top-gate or back-gate voltage. Hence, we conclude that weak localization or anti-localization is unlikely to be the origin of the focusing peak.

V. ROBUSTNESS OF FOCUSING PEAK POSITION AGAINST INJECTOR CONDUCTANCE

The occurrence of focusing peak is determined by the condition that the focal point of the lens aligns with the saddle point of QPC3, which is achieved by modulating N_r via tuning V_{lens}. The position of focusing peak should be insensitive to the status of the injectors because it does not affect N_r.

In the following experiment, as shown in Fig. S4, the conductance of the injectors was incremented from $0.8G_0$ up to channel fully open while the detector was fixed at G_0. The focusing peak always occurred around V_{lens} = -0.90 V until the QPCs entered the 1D-2D transition and 2D regime where injected electrons were no longer collimated[3].

On the other hand, the intensity of focusing peak ΔG, which was determined by the degree of electron collimation[4], was sensitive to injector conductance. It seemed that the focusing peak was initially strong, then significantly weakened when the injector conductance approached $3G_0$ and strengthened again at $5G_0$, as summarized by peak intensity ΔI_3 in Fig. S4(c). However, the non-monotonic behavior is because the competition between decreasing focusing efficiency [γ in S4(c)] and increasing total injection current I_{bak}] goes to Ohmic 3 with 1D channel open up. The reduction in γ suggested that the degree of electron collimation decayed with increasing conductance which was consistent with previous results[3,5].

VI. FOCUSING PEAK AS A FUNCTION OF TRANSVERSE MAGNETIC FIELD

The focusing process was closely associated with the electron trajectory. Therefore it was important to investigate the transverse magnetic field dependence of the focusing peak. As shown in Fig. S5(a), the focusing peak attenuated with an increasing magnetic field and eventually smeared out at a field of 30 mT. 30 mT corresponded to a cyclotron diameter of 5.6 μm which was larger than the distance between the injector and detector ($\sim$4.5 μm), therefore the focusing peak cannot be attributed to trivial geometric resonance[6]. Instead, the transverse magnetic field bent electron trajectory hence changed the incident angle with respect to the lens surface, and a large incident angle may cause total reflection and therefore prevent the occurrence of focusing peak. Similarly, the focusing peak smeared out at a higher magnetic field of 45 mT after the sample was illuminated by a red LED, as shown in Fig. S5(b).

It was also interesting to notice that residual peaks near the pinched-off regimes were far more robust against the magnetic field, which suggests that the origin for these peaks is different from the focusing peak. The residual peaks might be related to Coulomb blockade, due to the unintentionally formed constriction between the injector/detector QPCs and lens gate, which was known to be insensitive to small transverse magnetic field[7]; the Coulomb blockade interpretation may also account for the different temperature dependence of the residual peaks and focusing peak [Fig. 2 in main text].

VII. DETAILED DISCUSSION ON RELATION BETWEEN ELECTRON COLLIMATION AND FWHM OF FOCUSING PEAK

If the injected electrons have an angular spread within $\pm\beta$, electrons injected at $+\beta$ would be guided to f_2 on the primary axis and those injected at $-\beta$ would be guided to f_1 as depicted in Fig. S7, whereas electrons with other injection angles would end up in between. Similarly, electrons with smaller angular spread $\pm\beta^*$ distributes between f_2^* and f_1^*. The focusing peak starts forming when f_2 aligns with the entrance of detector (right FWHM point at less negative voltage end) of focusing peak. It is clear that f_2^* has not yet reached the entrance of detector at this gate voltage, therefore right FWHM point for smaller angular spread takes place at more negative gate voltage. On the other hand the focusing peak starts decaying when f_1 pass the exit of detector (left FWHM point at more negative voltage end); at the same gate voltage, f_1^* has already moved out of the channel, hence left FWHM point for smaller angular spread occurs at less negative gate voltage. Therefore, larger angular spread results in larger FWHM; equivalently speaking, larger FWHM indicates larger angular spread.

VIII. PROCEDURE OF SIMULATING CONFINEMENT WITH ASYMMETRIC GATE BIAS

First of all, we obtain the electrostatic confinement follows a standard framework[8] by simplifying the problem as the coupling between two semi-infinite metal stripes and 2DEG, while ignoring other factors such as dopant distribution. The obtained electrostatic confinement is then calibrated, in order to take screening into account, by inserting the confinement into saddle point model[9] to produce the conductance quantization, the calibration is achieved if the calculated position and length (with respect to gate voltage) of the conductance plateau matches the experimental result.

Plunging the calibrated electrostatic confinement U into Hamiltonian below, it then allows to obtain the electron distribution within the 1D channel and therefore the confinement profile with correction due to electron-electron interaction ,

$$H = \frac{p^2}{2m^*} + U + V_H + V_{exch}^{\sigma} \quad (1)$$

where p is the momentum and m^* is the effective mass,

Mode	E_b(meV)	W_{min}(nm)	W_{max}(nm)	α
Δ= 0 without e-e	8.575	77.8	185.1	5.245
Δ= 0 with e-e	8.490	78.2	185.0	5.771
Δ= -1 V without e-e	8.610	80.1	184.9	5.168
Δ= -1 V with e-e	8.372	80.5	184.9	6.772

TABLE II. Comparison between ΔV= 0 and -1 V.

U is the electrostatic potential, V_H is the Hartree interaction which takes the form[10],

$$V_H = \frac{e^2}{4\pi\varepsilon_0\varepsilon_r}\int dr' n(r')(\frac{1}{|r-r'|} - \frac{1}{\sqrt{|r-r'|^2+4b^2}}) \tag{2}$$

where the integral is performed over the whole area, ε_r and ε_0 are the relative dielectric constant of GaAs and dielectric constant of vacuum, respectively, $n(r')$ is the electron density, the term including b (the depth of the 2DEG) accounts for the mirror charge, and the exchange interaction takes the form[10] as

$$V^{\sigma}_{exch} = -\frac{e^2}{\varepsilon_0\varepsilon_r\pi^{3/2}}\sqrt{n^{\sigma}} \tag{3}$$

where σ denotes spin. Iteration stops until convergence is achieved. The calculated confinement potential and lateral electric field across the channel are presented in Fig. 4 of the main text.

IX. EFFECT OF ELECTRON-ELECTRON INTERACTION ON INJECTION ANGLE

For a symmetrically gate-biased QPC, the angle α within which the electrons are highly collimated is given by[11,12],

$$\alpha = \pm arcsin(\sqrt{\frac{E_F - E_b}{E_F}} \times \frac{W_{min}}{W_{max}}) \tag{4}$$

where E_b is the potential at the saddle point where minimum 1D channel width W_{min} occurs, W_{max} is the critical channel width where electron transport still remains non-adiabatic. Angular distribution for an asymmetrically gate-biased QPC has not been given explicitly in the literature; In order to make a comparison, we assume the same relation is still valid. Table II summaries the comparison between ΔV= 0 and -1 V with injector set to $\frac{2e^2}{h}$ ($E_F = 9$ meV).

It is necessary to comment that, the full angular distribution includes both the electrons within α (major contribution) and those in the tail (minor contribution) which can be obtained via Green′s function method. Usually the fully angular distribution follows the same trend as that of $\pm\alpha$[13].

The FWHM of the focusing peak is a function of both the angular distribution of injected electrons and length of the detector. However, the detailed form of the function is not known yet. For our particular experiment setup, it is rational to assume FWHM is proportional to the angular distribution of injected electrons because the length of the detector remains the same. It seems that a change in FWHM is observable only if we bring the e-e interaction into account.

* uceeya3@ucl.ac.uk

1 Thornton, T. J., Pepper, M., Ahmed, H., Davies, G. J. & Andrews, D. Universal conductance fluctuations and electron coherence lengths in a narrow two-dimensional electron gas. *Phys. Rev. B* **36**, 4514–4517 (1987).

2 Yamauchi, Y. *et al.* Universality of bias- and temperature-induced dephasing in ballistic electronic interferometers. *Phys. Rev. B* **79**, 161306 (2009).

3 Spector, J., Stormer, H., Baldwin, K., Pfeiffer, L. & West, K. Electron focusing in two-dimensional systems by means of an electrostatic lens. *Applied physics letters* **56**, 1290–1292 (1990).

4 Molenkamp, L. W., Brugmans, M. J. P., van Houten, H. & Foxon, C. T. Electron-electron scattering probed by a collimated electron beam. *Semiconductor Science and Technology* **7**, B228 (1992).

5 Sivan, U., Heiblum, M., Umbach, C. P. & Shtrikman, H. Electrostatic electron lens in the ballistic regime. *Phys. Rev. B* **41**, 7937–7940 (1990).

6 Duncan, D. S., Topinka, M. A., Westervelt, R. M., Maranowski, K. D. & Gossard, A. C. Aharonov-bohm phase shift in an open electron resonator. *Phys. Rev. B* **64**, 033310 (2001).

7 Ofek, N. *et al.* Role of interactions in an electronic fabry–perot interferometer operating in the quantum hall effect regime. *Proceedings of the National Academy of Sciences* (2010).

8 Davies, J. H., Larkin, I. A. & Sukhorukov, E. V. Modeling the patterned two-dimensional electron gas: Electrostatics. *Journal of Applied Physics* **77**, 4504–4512 (1995).

9 Martin-Moreno, L., Nicholls, J. T., Patel, N. K. & Pepper, M. Non-linear conductance of a saddle-point constriction. *Journal of Physics: Condensed Matter* **4**, 1323 (1992).

10 Wang, C.-K. & Berggren, K.-F. Spin splitting of subbands in quasi-one-dimensional electron quantum channels. *Phys. Rev. B* **54**, R14257–R14260 (1996).

[11] Beenakker, C. W. J. & Houten, H. v. Magnetotransport and nonadditivity of point-contact resistances in series. *Phys. Rev. B* **39**, 10445–10448 (1989).

[12] Crook, R., Smith, C. G., Barnes, C. H. W., Simmons, M. Y. & Ritchie, D. A. Imaging diffraction-limited electronic collimation from a non-equilibrium one-dimensional ballistic constriction. *Journal of Physics: Condensed Matter* **12**, L167–L172 (2000).

[13] Saito, M., Takatsu, M., Okada, M. & Yokoyama, N. Analysis of the angular distribution of electrons injected through a quantum point contact by use of a green's function with a weak-magnetic-field approximation. *Phys. Rev. B* **46**, 13220–13233 (1992).
